# Defining Insights

**DOI:** 10.1007/s43441-023-00554-w

**Published:** 2023-07-05

**Authors:** Alicia A. Cadogan, Jerry Lau, Susan Wnorowski, Geri R. Kelsch, Jane Oreper, Lillian Chavez, Joseph J. Weidman, Evelyn R. Hermes-DeSantis

**Affiliations:** 1 Pfizer Inc, New York, USA; 2 Rutgers The State University of New Jersey, New Brunswick, USA; 3 Ipsen Biopharmaceuticals, Inc., Basking Ridge, USA; 4 Genmab US, Inc., Princeton, USA; 5 Novartis Pharmaceuticals Corporation, East Hanover, USA; 6 Takeda Pharmaceuticals USA Inc, Deerfield, USA; 7 phactMI, 142 Glen Mills Rd., PO Box 23, Glen Mills, PA 19342 USA; 8 Professor Emerita, Rutgers, The State University of New Jersey, New Brunswick, USA

**Keywords:** Insights, Medical information, Medical affairs, Pharmaceutical industry, phactMI, Metrics

## Abstract

**Background:**

Insights, when acted upon, can result in positive changes to the business, for HCPs, and ultimately for patients. Medical Information, as a customer facing function, is one of the groups that generate insights. Data and insights across different functions of an organization need to be compiled to provide a comprehensive view. The purpose of this paper is to develop a shared definition of insights and to provide a working guidance for the insight process.

**Methods:**

Two surveys were conducted of the phactMI membership first to establish a shared definition of insights and then to benchmark current insight process. From this data and the shared experience of the working group a proposed guidance was developed.

**Results:**

The developed definition of an insight is “An insight is the deeper understanding of the why behind trends of information that lead us to determine if an action is warranted”. For the most robust outcomes, insight identification needs to be a cross functional activity. The proposed structured approach can be leveraged and customized for any organization and include the following five steps: INvestigate, Scrutinize, Identify, Take Action, and Enlighten (INSITE).

**Conclusion:**

The INSITE process provides a simple framework that should become routine for all Medical Information colleagues who are leading the work around insights. The process should be shared across all functions that participate in the insight generation process. This is another area where Medical Information can demonstrate leadership and highlight their value to the organization.

**Supplementary Information:**

The online version contains supplementary material available at 10.1007/s43441-023-00554-w.

## Introduction

Medical information is a customer facing role where health care professionals (HCPs), consumers, and others request product-specific information from the pharmaceutical company [1]. Over time each company has access to a robust database of inquiries and when sorted and analyzed appropriately, Medical Information is in a unique position to identify important and meaningful trends. When looking beyond the numbers and trends, we can then identify insights that, when acted upon, can result in positive changes to the business, for HCPs, and ultimately for patients. Such action will not happen without effort. Identifying meaningful insights and following through with action requires an intentional approach that is coordinated by colleagues who are committed to seeing the impact and value that this effort can produce [2].

When embarking on the journey to identify and act upon insights it is critical that there is a clear intent to be more customer-centric [3]. Clarity is needed around what constitutes an insight, who is responsible for identifying and acting on insights, the processes required to streamline this important deliverable, and the technology needed to support the efforts. When the phactMI working group began discussing the topic of insights it became evident there were more questions than answers. Our discussions centered on some common themes, and we sought to answer some very basic questions about insights.

### Definition of Insights

The Merriam-Webster dictionary defines “insight” as “(1) the power or act of seeing into a situation or (2) the act or result of apprehending the inner nature of things or of seeing intuitively.” (https://www.merriam-webster.com/dictionary/insight) The application of that definition in the business realm is not straight forward. Within each company, it is likely that each individual has their own specific definition of an insight. A common definition of an insight that is centered on the work conducted in Medical Information can allow stakeholders to move towards a common strategic endpoint in support of the needs of HCPs and patients.

There are many external-facing groups within pharma that can compile information and share metrics and trends based on their observations [4]. However, metrics are very different from insights. There is a difference between graphing the number of questions on a particular topic and understanding why the question is being asked and what action should be recommended to help support those asking the question [5]. Similarly, there is a difference between reporting which segment of HCP customers contact Medical Information most frequently and developing an action plan to address the unique needs of that customer segment. While both metrics and insights are important and help to inform the business, insights are often supported by metrics and require action to make an impactful change. Customer insights include learning about human behavior and their underlying motivations behind the behavior. Insights may also be considered information that challenges what we believe about our customers and drives us towards seeing our customers in a new way [5]. Ultimately the reason we identify insights and act upon them is to improve processes, or medical strategies, enhance customer’s experience, or to have more data to support appropriate business decisions focused on improving outcomes for patients. With fully developed insights, we can understand the current needs and better prepare for future needs [6].

Insight identification can be conducted by many different departments. Typically, Medical Information, Field Medical, Medical Affairs, Safety, Health Outcomes, and others can, and often do, identify insights. Most often this work is done independently within each department and is derived from their function-specific databases. However, in the ideal world, data across different parts of an organization can be compiled to provide a comprehensive view of all the available data in the enterprise. This would result in a decision that is based on a more holistic view.

Insight identification is handled differently from company to company. Some variations include which function within the company leads the effort, the scope of insights (local, regional, global), and whether data from different departments are combined when identifying insights. All these variables can impact the validity of the observations and can affect the overall recommendation(s) and resultant action(s).

The actual process of identifying insights is on a spectrum ranging from a tedious manual exercise to one supported heavily by technology. When the task is manual, it can deter the organization from putting resources behind it; however, developing technology solutions can be costly. Regardless of where each company lands on this spectrum, a professional with extensive product knowledge must be part of the process to bring clarity and reality to the observations.

With all these variables it became clear that the industry could benefit from an overarching framework around insights. The purpose of this paper is to develop a shared definition of insights and to provide a working guidance for the insight process.

## Materials and Methods

In order to understand how insights are identified and acted upon in the pharmaceutical industry and what role Medical Information plays in the process a survey was developed to gather information directly from the phactMI member companies. When developing the survey, it became clear there were different interpretations of what constitutes an insight. Therefore, to first address the need for a common definition of the word ‘insight’ as it applies to the industry a questionnaire was developed. A 5-question survey was sent to the 31 member companies asking if their company and/or Medical Information Department had a definition of an insight that they could share, and what sources they utilized to identify insights.

The responses were compiled, and a word cloud approach was used to derive a definition that encompassed the voice of the 19 responding companies. With this as a foundation the working group developed a 21-question survey digging deeper into how companies identify, act upon, and communicate insights. The results from the 15 responding companies are described within this report and provide the foundation for the recommendations.

## Results

**Figure 1:**
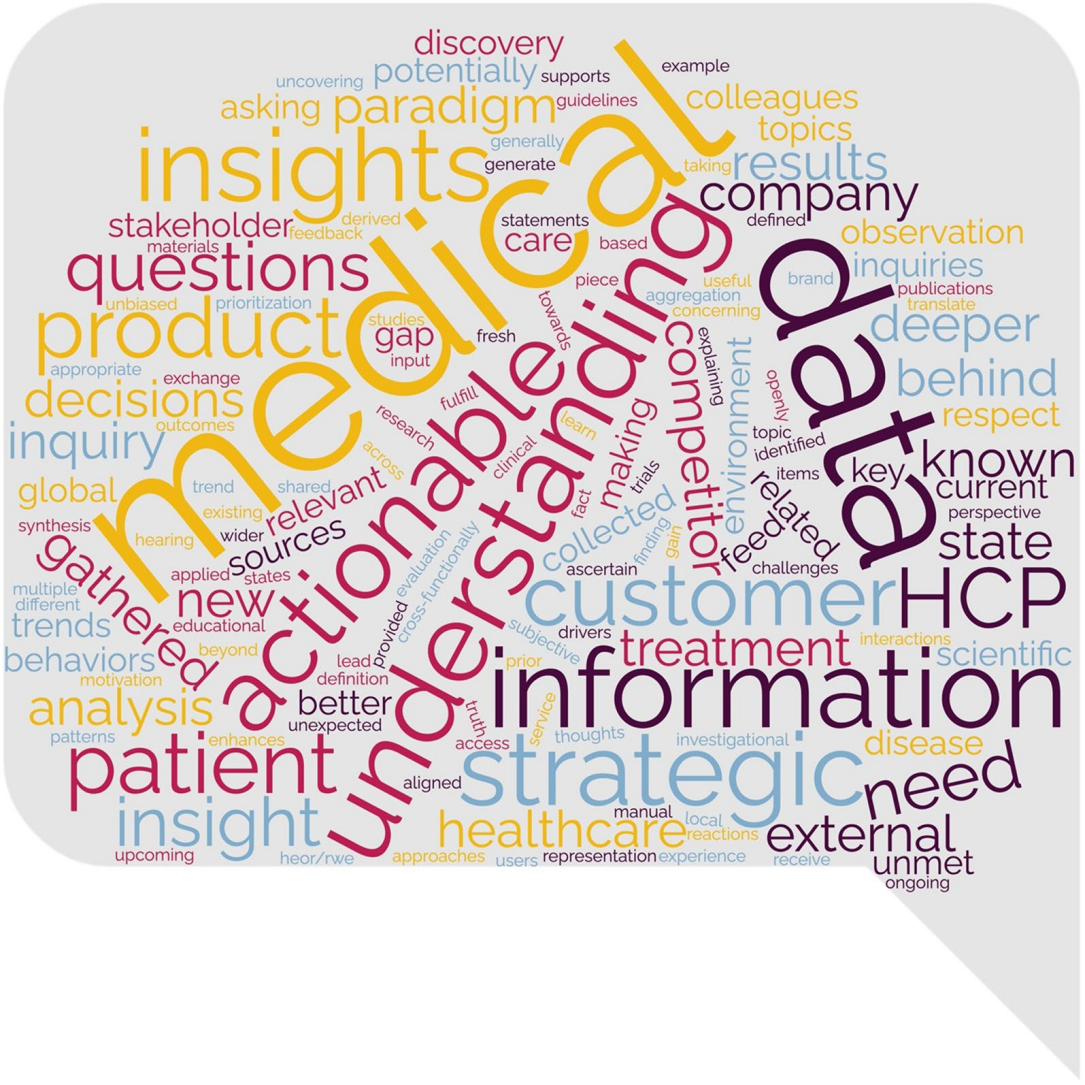
Word Cloud from Insight Descriptions.

Based on responses from the initial survey, a word cloud was constructed of important terms for the definition of an insight. (See Fig. 1).

From the word cloud and associated definitions provided, the working group developed the following definition. Key take-aways from the definition are that an insight is more than just data, it is trends that cause this data to rise to the top; and, while many say that insights must be actionable, this group recognized that some insights are confirmatory and therefore no further action is necessary.

**Table:** 

“An insight is the deeper understanding of the why behind trends of information that lead us to determine if an action is warranted”	

### Collaborations/Partners

**Figure 2:**
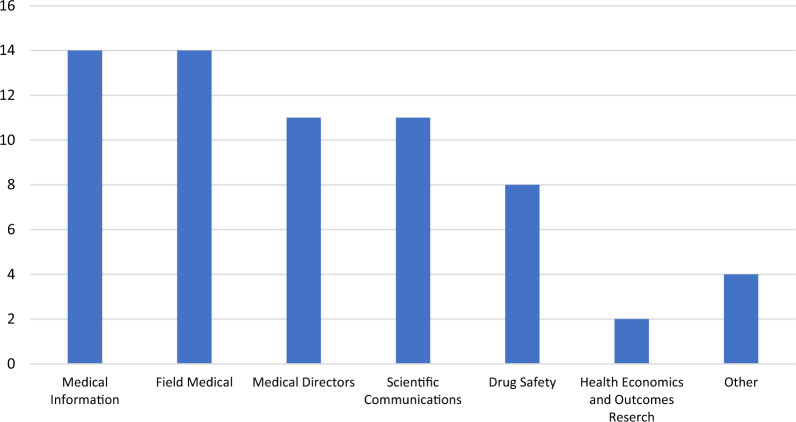
Functions Involved in Insight Identification (*n* = 15). Other Includes Medical Insights Team Collaboration, Patient Advocacy, Policy, and Labeling/Manufacturing.

It is important to know the different functions within a company that identify insights to help understand the perspective from which the insight comes and who is responsible for insight identification. Insights from Medical Information could be different from those of Safety, Field Medical, or Health Outcomes. If all these functions identify insights independently there could easily be an opportunity for collaboration. The findings revealed that in most companies more than one function identifies insights (Fig. 2). Medical Information and Field Medical were selected as identifying insights by 93% of respondents, Medical Directors and Scientific Communications by 73%; Drug Safety, 53%; Health Outcomes, 13%, and 7% selected each of the following: Medical Insights Team Collaboration, Patient Advocacy, Policy, and Labeling/Manufacturing. It is evident that in many companies there are several functions that make insight identification a priority.

Since so many functions within a company identify insights, it is important to understand if and how they work together with Medical Information on this task. Overall, 40% of Field Medical (6/15) collaborate with Medical Information on insight identification. The collaboration rate with Medical Information was 33% with Medical Affairs/Medical Operations and 27% with Medical Directors. The list of collaborators is extensive (Table 1) which indicates that insight identification is not, nor should be, an independent activity. In order to identify deep and meaningful insights, the functions within pharma need to work together for clarification, understanding, and confirmation to effect change.

**Table 1:** Functions that Medical Information Collaborates with to Identify Insights (*n* = 15).

Function	Number of Companies
Field medical/medical science liaisons	6
Medical affairs/medical operation	5
Medical directors	3
HEOR	3
Commercial/promotional	2
Medical/scientific communications	2
Drug safety	1
Labeling	1
Manufacturing	1
Patient advocacy	1
Policy	1
Regulatory	1

Regardless of who identifies insights, it is rare that the next steps will be independent. When insights are identified they typically require internal sharing so that they can be acted upon. This includes confirming the validity of the finding and agreeing on next steps to improve a process or an experience as described previously. When asked which functions Medical Information shares their insights with the majority indicated Medical Affairs (100%) and Field Medical (80%). A smaller segment (60%) shared with Marketing and Health Outcomes, and fewer (27% or less) shared with Drug Safety and others. Sharing insights can serve several different purposes. Whether sharing for informational purposes, seeking support in validating observations, or looking for collaboration with next steps in the process, it is important to identify and partner with the right business partners when working on insights.

Collaborating with other functions to identify insights is a common practice amongst Medical Information teams. Overall, 87% (13/15) of Medical Information teams collaborate with other functions to identify insights (including joint review of data or having a discussion to validate a possible insight). It was not clear from this survey if there were formal processes around how this collaboration occurred (such as frequency, documentation, or who leads the process).

### Identification/Categorizing Insights

It is important to understand how broad the scope of insights is at each individual company. Based on this survey, 60% (9/15) of companies identify and act upon insights at the regional level, while 27% (4/15) are at the global level and only 13% (2/15) are solely at the local level.

Most companies, 73% (11/15) do not have a standard operating procedure or guidance on identifying insights, which can lead to different interpretations across the organization.

While all the respondents agreed that identifying insights is considered a strategic initiative, it was only considered strategic at the company level for 40%. The majority, 60%, indicated it was only a strategic initiative at the group/department level.

When asked to categorize the potential areas a company focuses on when identifying insights, it was either an educational gap or safety signal (73% for both), followed by competitive information (60%), development information (53%), and product life cycle management (53%). Frequently asked questions or Medical Information content needs only consisted of 33% of insights and emerging trends were only 7% of the focus.

A common thread in several responses was having one system or at least working in cross-functional teams to identify insights to avoid duplication and assist with potential action triage. Other recommendations included utilizing artificial intelligence and critical analysis to assist in understanding the drivers of trends and patterns. An additional thought was emphasizing the strategic role that Medical Information can have at the forefront of the cross-functional process.

A majority, 87% of respondents did not have a best practice in insight identification to share. However, two companies shared the following best practices based on their experiences:

- “Leveling up by asking probing questions to convert an observation to an insight; train colleagues to recognize differences; and how to gather and report insights ethically and responsibly.”
- “Is the unbiased representation of cross-functionally collected customers’ input resulting from aggregation, understanding and prioritization. That informs action and strategic decisions across [the company] towards making patient care better.”

### Analyzing Insights

**Figure 3:**
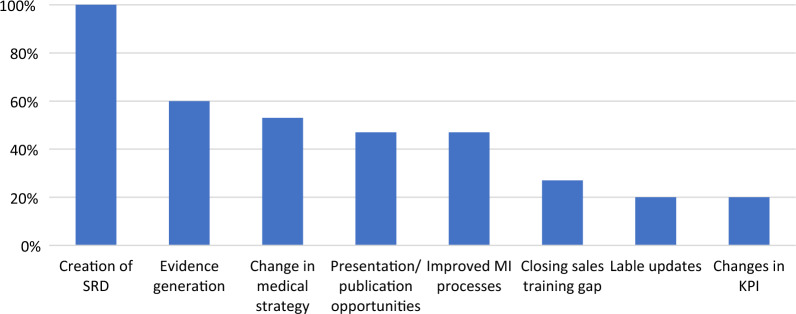
Actions Taken Based on Insights.

After developing a working definition of insights and better understanding the process used by companies for identifying them, the next step was to determine if there was a correlation between the insights collected and the type of actions that resulted. This is important as it may allow organizations to be more responsive to external conditions. Our survey uncovered that from a Medical Information perspective, all responding companies reported that insights led to the creation of new scientific response documents, 60% resulted in evidence generation, 53% led to a change in their medical strategy, and 47% led to presentation/publication opportunities and improved Medical Information processes within their companies. It also revealed that 27% of respondents experienced a closing in their sales training gap; 20% led to label updates and changes as well as changes in measures of key performance indicators. (see Fig. 3).

There is no consistent way of measuring the impact of insights as is evidenced by survey results. Overall, 40% (6/15) of respondents did not measure the impact of insights, 40% responded they measured the impact of insights, and 20% (3/15) did not respond to the question. Table 2 summarizes the open-ended responses concerning measuring the impact of insights.

**Table 2:** Measuring Impact of Insights.

Impact of Insights Measurements	Reasons for Not Measuring Impact
• Decrease in escalation in medical information inquires and inquiry volume received	• Unaware of how to measure
• Number of inquiries received which are related to insights	• No formal system in place
• What changed, what improved, and value added	• No effective tool/tracker to quantify the impact of insights

### Technology

Amongst the 15 companies surveyed, 2 utilized the same technology as their collaborators for identifying insights while 11 used different approaches. Reasons for using dissimilar technologies included: different systems (*n* = 5), unknown/unsure (*n* = 2), a lack of necessity (*n* = 1), Medical Information collecting insights and sharing with the broader Medical team (*n* = 1), insights tool solely accessible to Field Medical teams (*n* = 1), and development of technology was underway (*n* = 1).

Respondents were asked to list the technologies and/or software used by their function to collect insights. Fourteen of the companies utilized commercially available technology. Other types of technology listed were “no specific technology/none” (*n* = 4), a home-grown system (*n* = 1), and unknown (*n* = 1). Participants who listed a specific software most reported use of Salesforce (*n* = 4) while there was one mention each for 3Vue, Adobe Analytics, Business Intelligence Platform, Geodart, IRMS-DV, Luminoso, Microsoft, Qlik Sense, Spotfire, Stratifyd, Tableau, Veeva, and X-Fly.

Utilization of technology by the survey recipients varied throughout the insight identification process. Technology was most frequently used for data analysis (60%; *n* = 9) followed by input of insights (40%; *n* = 6), communication of findings or subsequent action (27%; *n* = 4), and to pull reports for manual comparison (7%; *n* = 1). Two respondents (13%) did not use any technology throughout the insight identification process.

### Overall Process

A total of 53% (8/15) of respondents felt the insight process within their company was effective and 47% (7/15) of respondents felt it was not. Table 3 summarizes examples of effective insight processes that were provided, and Table 4 provides potential improvements to the insight process based on survey responses and interpretation of the data set overall.

**Table 3:** Effective Insight Process—Examples.

• Process succeeded in allowing them to create and communicate insights	
• Provides topic of interests for products	
• Provides the ability to collaborate across multiple functions	
• Insights can be relayed to leadership for action	
• A dedicated function to oversee insight submission and dissemination

**Table 4:** Potential Improvements to the Insight Process.

Survey Responses	
• in a manual process, the development of training and resources to help with insight identification could be useful to gain a better understanding of the organization's work/strategies so MI insights can inform tactics	
• developing a harmonized method of data capture and the implementation of robust tools to identify and help the initial analysis of insights	
• the addition of a technology platform to automate or better manage the manual elements	
• the development of one system to collect and analyze insights with relevant groups	
Additional recommendations	
• One system or at least working in cross-functional teams to identify insights to avoid duplication and assist with potential action triage	
• utilizing artificial intelligence and critical analysis to assist in understanding the drivers of trends and patterns	
• emphasizing the strategic role that Medical Information can have at the forefront of the cross-functional process	

All 15 respondents felt that their organization would benefit from a formal structure in insight identification and communication. When asked where there were opportunities for Medical Information to be involved in the development of insights, responses included:

- the use of effective tools to help assess data and derive actionable insights would help Medical Information to be able to share information with other groups and improve resources available for HCPs and patients
- as a reactive facing role who receives information across varying TA/products we are in a good position to collect, analyze, share and collaborate with relevant groups
- provide proactive and timely communication
- connect Medical Information insights to plan of action and strategic plan
- have the ability to identify trends from inquiries,
- Medical Information is on the forefront to play a strategic role,
- develop a structured approach for standardizing Medical Information insights so they are routinely identified,
- document insights and interactions with both the contact center, field and customers to help assess appropriate actions and prioritizations,
- the ability to raise questions with no available standard responses immediately up to strategic points in Medical Affairs so it could be analyzed and actions could be determined, and
- access insights for regular reporting where suggestions can be made for new response documents.

Clearly there is opportunity here to uncover, learn, and provide best practices for identifying, socializing, and developing metrics to quantify the impact of measuring insights.

## Discussion

The survey findings revealed that a formal process for identifying and communicating insights is needed and would be a valuable tool for Medical Information. Standardizing a specific process across companies poses challenges for various reasons, one being the uniqueness of each organization's composition and reporting structure. However, the structured approach below can be leveraged and customized for any organization and include the following five steps: INvestigate, Scrutinize, Identify, Take Action, and Enlighten.
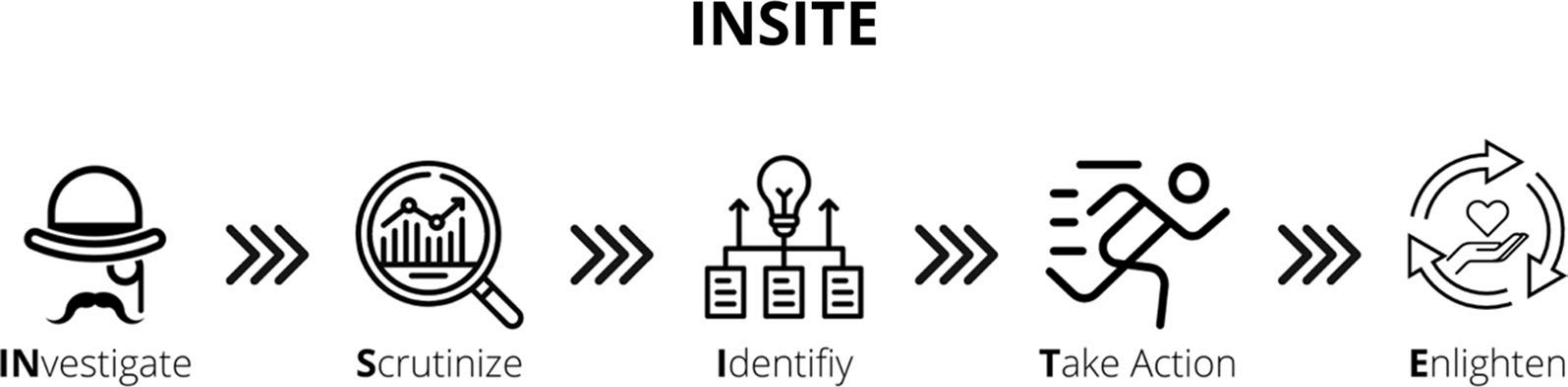


## INvestigate

This first step starts with investigating and gathering data that provides a view into what customers are saying. This can, and should, include data from Medical Information, Field Medical, Health Outcomes, Medical Directors, and other individuals/departments who engage with external healthcare professionals (HCPs). Although insights can more easily be identified from one data source, consolidating input from various departments will generate a more comprehensive view of the experience with a particular product. Numerous tools can support this consolidation, from Excel spreadsheets to artificial intelligence.

## Scrutinize

Technological support is critical at this stage because the volume of data is often too great to manage without its utilization. Medical Information expertise is needed to scrutinize and determine the appropriate filters and categories that will provide data in a format that is easily combed through by a Medical Information professional, and technology will make this process much more manageable. In addition, the available resources for oversight and budget may determine what works best for each organization. Ultimately, this step involves taking a deeper look at the existing data and making those insightful observations.

## Identify

Once the technology shapes the information into a manageable format, the human element must take the lead. It takes deep product knowledge and an understanding of the target customer(s) to parse through the many interesting observations and trends in the data to identify meaningful insights. The "filtering" of the investigated and scrutinized data is critical because it can potentially alter trends in business approaches and better patient outcomes. We want to be certain that when the information comes into focus our interpretation is accurate.

## Take Action

Once an actionable insight is identified, it must be acted upon promptly. The nature of the insight should dictate who can take action. Providing the insights to the appropriate colleagues ensures that the right stakeholders have sufficient information and situational understanding to drive change and facilitate a meaningful outcome. For example, if the action is to create a new scientific response document, then Medical Information will drive that forward. However, if a labeling change is required, communication with our medical colleagues is necessary for additional support/validation and collaboration to present our findings to Labeling who can then take the next steps. Collaboration across functions is highly encouraged for both validation and support especially when the necessary action is beyond the scope of Medical Information.

## Enlighten

It cannot be stressed enough that to enlighten colleagues on the outcome of insights that were identified and acted upon is a critical step in this process. Communicating the value of the efforts of dedicated individuals in Medical Information and other departments can justify the time and effort required and garner additional support. In addition, it highlights the importance of this necessary work and the contribution that Medical Information brings to the process.

Focusing on insights requires a commitment of resources. At a time where every company has limited resources and is faced with the need to justify budgets it is important to be able to demonstrate not only what was done, but also the value that this investment will return. This may require tracking the actions that were taken based on the identified insights and determining if the value could be attributed to one of the following categories:

- Time
  - Creating a new scientific response document saved time that Medical Information would take to research and create a custom document each time the same question is asked
- Patient Impact
  - Change in label provided clarity for prescribers and minimized the risk of unsafe product use
- Resources
  - Collaborating on insights instead of working in silos minimized effort duplication and free up resources for other activities

When enlightening others of the good work that was done it would be a good practice to include this context.

## Conclusion

Medical Information colleagues can bring value to their organization by leveraging their external facing role and the to look inward and analyze their rich inquiry databases to understand the why behind customer inquiries and the potential subsequent actions that they can take. These insights can change how HCPs receive, process, and utilize information to make treatment decisions. Every company talks of insights; however, without a common definition there could be confusion or missed opportunities. The working definition offered in this paper should be socialized within companies to ensure that everyone’s thinking is aligned to the process and desired outcomes. In situations where the term insight is being used outside of this definition (e.g. referring to metrics) we encourage you bring clarity to the conversation.

The INSITE process offered here provides a simple framework that should become routine for all Medical Information colleagues who are leading the work around insights. The process should be shared across all functions that participate in the insight generation process. When used along with the proper definition of an insight, one can expect their efforts to be more efficient, and that there will be more opportunities for collaboration across functions. Additionally, the impact related to time, patient impact, or resources should be included when enlightening business partners about the output of these efforts. Stepping in the forefront of the insight process takes courage and requires support from the organization. But it is well worth the effort and provides yet another way that Medical Information can demonstrate leadership and highlight their value to the organization.

### Supplementary Information

Below is the link to the electronic supplementary material.

Supplementary file1 (DOCX 19 KB)

## Data Availability

The datasets analyzed for this study are available from the corresponding author upon a reasonable request.
