# Supplementary material for: Defining Insights
Source: Ther Innov Regul Sci. 2023 Jul 5;57(6):1229–37. doi: 10.1007/s43441-023-00554-w (PMC10579153; doi:10.1007/s43441-023-00554-w)
Supplement: Supplementary file 1 — Supplementary file1 (DOCX 19 KB) [file 43441_2023_554_MOESM1_ESM.docx]

**Defining Insights part 1**

Please respond based on how you handle a newly (within the last 5 years) marketed product.

1. How do you define an insight?
2. How does your company/medical affairs organization define an insight?
3. What are the sources of insights? (select all that apply)
   1. Medical Information
   2. Field Medical
   3. Social Media
   4. Advisory Board meetings
   5. Speaker Training
   6. Clinical investigators
   7. Other (please specify)
4. Does your company differentiate between insights and data/observation/metric/activity reports?
   1. Yes
   2. No

**Defining Insights – part 2 – The process**

For this survey, we have developed the following working definition of a survey based on phactMI member input:

**An insight is the deeper understanding of the why behind trends of information that lead us to determine if an action is warranted**

Please respond based on how you approach insights with a product marketed within the last 5 years.

1. Who identifies insights within your company? (select all that apply)
   1. Medical Information
   2. Medical Director
   3. Field Medical
   4. Drug Safety
   5. Other (please specify)
2. Does Medical Information in your company collaborate with other functions in identifying insights? (Examples of collaboration include reviewing data/reports or having discussions to validate that an insight was identified)
   1. Yes, please list the functions (go to question 4)
   2. No (please explain)
3. Do the functions that you collaborate with utilize the same technology as Medical Information?
   1. Yes (go to question 5)
   2. No (explain why not) (go to question 6)
4. Please list the technology/software that you use.
5. Please indicate where you use technology in the insight identification process
   1. Analysis of data
   2. Communication of findings or subsequent actions
   3. Other, please specify
6. At which level are insights identified and acted upon? (select all that apply)
   1. Country
   2. Region
   3. Global
7. Does Medical Information have a Standard Operating Procedure on identifying insights?
   1. Yes
   2. No
8. Is identifying insights considered a strategic initiative?
   1. Yes, at the group/department level only
   2. Yes, at the company level
   3. No (explain why not)
9. How frequently do you go through the process of identifying and sharing insights? (select all that apply) (build as matrix)
   1. Scheduled weekly
   2. Scheduled monthly
   3. Scheduled quarterly
   4. On an as needed basis
   5. Other
10. What categories do you focus on for insight identification? (select all that apply)
    1. Competitive information
    2. Development information
    3. Product life cycle management
    4. Educational gaps
    5. Safety signals
    6. Other (please specify)
11. What type of actions has resulted from the identification of insights?
    1. Generate/create new Medical Information letter
    2. Evidence generation
    3. Presentation/publication opportunity
    4. Change in KPI measure
    5. Improving MI processes at a particular company
    6. Close a gap in sales training
    7. Change in medical strategy
12. Do you track the actions taken as a result of insights identification?
    1. Yes (please explain)
    2. No
13. How do you measure the impact of the insight? Free text
14. Which function receive insights identified by Medical Information? (select all that apply)
    1. Medical affairs
    2. Field medical
    3. Drug safety
    4. HEOR
    5. Medical outcomes specialists
    6. Marketing
    7. Other (please specify)
15. Do you have a best practice in insight identification that you are willing to share with PhactMI membership?
    1. Yes, please specify
    2. No
16. Overall, do you feel that the insight process in your company is effective?
    1. Yes, please explain
    2. No
17. What changes would you make?
18. Would the organization benefit from a formal structure in insight identification and communication?
19. Where are the opportunities for Medical Information to be more involved in identifying insights?
20. Is there a better way to identify insights?
